# Supplementary material for: Are Undernutrition and Obesity Associated with Post-Discharge Mortality and Re-Hospitalization after Hospitalization with Community-Acquired Pneumonia?
Source: Nutrients. 2022 Nov 19;14(22):4906. doi: 10.3390/nu14224906 (PMC9697837; doi:10.3390/nu14224906)
Supplement: Supplementary file 1 [file nutrients-14-04906-s001.zip › nutrients-1952284-supplementary.pdf]

Supplementary Table S1. Characteristics during hospital admission and outcome measures among eligible and ineligible patients hospitalized with community-acquired pneumonia.

	Eligible patients (n=323)	Ineligible patients (n=397)	p-value
Age, years	69.8±13.6	70.0±15.2	0.78
Female sex	156 (48.3)	192 (48.5)	0.96
**Comorbidities**			
Charlson Comorbidity index	4.3±2.5	4.4±2.8	0.81
**Laboratory characteristics**			
C-reactive protein, mg/L	97.3 (35.8-159.7)	108.2 (47.2-180.7)	0.07
**Severity characteristics**			
CURB-65			0.23
Mild	161 (57.1)	188 (51.5)	
Moderate	95 (33.7)	130 (35.6)	
Severe	26 (9.2)	47 (12.9)	
Length of stay, days	5.5 (3.4-9.0)	5.2 (3.3-8.5)	0.70
ICU admission	8 (2.5)	18 (4.7)	0.13
**Outcomes**			
**Re-hospitalization**			
30-day	74 (22.9)	87 (21.9)	0.75
90-day	97 (30.0)	110 (27.9)	0.52
180-day	129 (39.9)	155 (39.0)	0.81
**Mortality**			
30-day	11 (3.4)	18 (4.5)	0.44
90-day	25 (7.7)	42 (10.6)	0.19
180-day	38 (11.8)	54 (13.9)	0.40

Patients were considered eligible if they were not participating in our physical training intervention or if they had sufficient data to answer the research question (categorizing into relevant groups). Differences between groups were evaluated using t-test and Kruskal Wallis test for normal distributed variables and non-normally distributed variables, respectively. For categorical variables Pearson chi-square tests were used. Data are presented as median (IQR), mean ±SD and n (%). P-values < 0.05 are considered significant.

Supplementary Table S2. Differences in characteristics during admission among undernourished, well-nourished, overweight, and obese patients hospitalized with community-acquired pneumonia.

	Well-nourished vs. undernourished	p-value	Well-nourished vs. overweight	p-value	Well-nourished vs. obese	p-value	Overweight vs. under-nourished	p-value	Overweight vs. obese	p-value	Underweight vs. obese	p-value
Age, years	-5.9 (-11.8; 0.02)	0.051	-0.2 (-5.2; 4.8)	1.00	3.5 (-2.0; 9.0)	0.35	-5.7 (-11.3; -0.05)	0.047	3.7 (-1.5; 8.9)	0.25	-9.4 (-15.4; -3.4)	<0.001
Female sex	-14 (-37; 8)	0.23	20 (-1; 41)	0.07	-8 (-30; 4)	0.48	-20 (-42; 2)	0.08	-14 (-35; 7)	0.20	6 (-16; 28)	0.60
BMI, kg/m^2	3.3 (2.1; 4.5)	<0.001	-4.5 (-5.5; -3.5)	<0.001	-12.2 (-13.3; -11.1)	<0.001	7.7 (6.6; 8.9)	<0.001	-7.7 (-8.7; -6.6)	<0.001	-15.4 (-16.7; -14.2)	<0.001
Fat-free mass index, kg/m^2	2.0 (1.0; 3.0)	<0.001	-1.6 (-2.5; 0.8)	<0.001	-3.7 (-4.6; 2.8)	<0.001	3.6 (2.7; 4.5)	<0.001	-2.1 (1.2; 2.9)	<0.001	-5.7 (-6.7; 4.7)	<0.001
**Comorbidities**												
Charlson comorbidity index	-0.6 (-1.7; 0.5)	0.54	0.7 (-0.2; 1.6)	0.22	0.7 (-0.3; 1.7)	0.32	-1.3 (-2.3; -0.2)	0.01	0.02 (-0.9; 1.0)	1.00	2.3 (-0.1; 2.4)	0.02
**Laboratory characteristics**												
C-reactive protein, mg/L	35 (-60; 8)	0.13	-15 (-75; 25)	0.84	-62 (-56; 38)	1.00	-25 (-53; 21)	0.41	86 (-33; 45)	0.80	36 (-7; 61)	0.12
**Severity characteristics**												
CURB-65 index												
Mild	11 (-19; 41)	0.52	-4 (-28; 20)	0.75	-8 (-35; 19)	0.56	15 (-14; 44)	0.39	-4 (-29; 21)	0.76	-19 (-50; 12)	0.31
Moderate	-3 (-21; 15)	0.75	5 (-12; 22)	0.55	11 (-8; 30)	0.25	-8 (-26; 10)	0.39	6 (-12; 24)	0.52	14 (-6; 34)	0.18
Severe	-8 (-40; 24)	0.65	-1 (-29; 27)	0.95	- 3 (-33; 27)	0.85	-7 (-38; 24)	0.66	-2 (30; 26)	0.89	5 (-27; 37)	0.76
Length of stay, days	6 (-33; 33)	0.97	8 (-23; 31)	0.89	- 3 (-34: 29)	1.00	-13 (-37; 21)	0.69	-6 (-22; 42)	0.97	-8 (-30; 35)	0.92

Differences between groups were evaluated using Pearson chi-square tests presented as absolute difference in percentage points for categorical variables and oneway ANOVA for continuous variables presented as absolute differences or relative percentage differences with 95% confidence intervals and p-value for normally and non-normally distributed variables, respectively. Variables were log-transformed as appropriate.

Supplementary Table S3. Comorbidities among undernourished, well-nourished, overweight, and obese patients hospitalized with community-acquired pneumonia.

	**Total sample (n=323)**	**Undernourished (n=57)**	**Well-nourished (n=83)**	**Overweight (n=107)**	**Over-nourished (n=76)**
**Comorbidities**					
COPD	112 (34.7)	33 (57.9)	27 (32.5)	32 (29.9)	20 (26.3)
Cardiovascular diseases	95 (29.4)	19 (33.3)	22 (29.9)	32 (29.9)	22 (29.0)
Cancer	56 (17.3)	16 (28.1)	16 (19.3)	13 (12.2)	11 (14.5)
Diabetes	50 (15.5)	7 (12.3)	10 (12.1)	15 (14.0)	18 (23.7)
Data shown as n (%).					

Supplementary Table S4. Differences in comorbidities among undernourished, well-nourished, overweight, and obese patients hospitalized with community-acquired pneumonia.

	**Well-nourished vs. undernourished**	**p**	**Well-nourished vs. overweight**	**p**	**Well-nourished vs. obese**	**p**	**Overweight vs. under-nourished**	**P**	**Overweight vs. obese**	**P**	**Underweight vs. obese**	**p**
Chronic obstructive pulmonary disease	-25 (-49; -0.01)	0.053	3 (-21; 27)	0.80	7 (-19; 33)	0.60	-28 (-51; -5)	0.02	4 (-21; 29)	0.76	32 (6; 58)	0.02
Cardiovascular diseases	-3 (-32; 26)	0.84	0 (-25; 25)	1.00	1 (-24; 26)	0.94	-3 (-29; 23)	0.82	1 (-24; 26)	0.94	4 (-24; 32)	0.78
Cancer	-9 (-38; 20)	0.54	7 (-19; 33)	0.61	4 (-25; 33)	0.79	-16 (-0.44; 12)	0.29	-3 (-31; 25)	0.83	13 (-17; 43)	0.43
Diabetes	0 (-31; 31)	1.00	-2 (-29; 25)	0.88	-12 (-40; 16)	0.44	2 (-28; 32)	0.90	-10 (-36; 16)	0.47	-12 (-43; 19)	0.51

Differences between groups were evaluated using Pearson chi-square tests presented as absolute difference in percentage points for categorical variables and oneway ANOVA for continuous variables presented as absolute differences or relative percentage differences with 95% confidence intervals and p-value for normally and non-normally distributed variables, respectively. Variables were log-transformed as appropriate.

Supplementary Table S5. Characteristics during hospital admission and outcome measures among undernourished patients hospitalized with community-acquired pneumonia.

	Patients with BMI <18.5 kg/m2 (n=15)	Patients with BMI >18.5 plus weight loss (n=42)	p-value
Age, years	72.4±10.9	76.5±13.7	0.30
Female sex	12 (80.0)	22 (52.4)	0.06
**Comorbidities**			
Charlson Comorbidity Index	4.7±1.5	5.3±2.4	0.37
COPD	14 (93.3)	19 (45.2)	0.001
Cardiovascular diseases	2 (13.3)	17 (40.5)	0.07
Cancer	6 (40.0)	10 (23.8)	0.23
Diabetes	1 (6.7)	6 (14.3)	0.44
**Laboratory characteristics**			
C-reactive protein, mg/L	126.7 (87.2-138.2)	132.6 (52.2-183.4)	0.50
**Severity characteristics**			
CURB-65			
Mild	3 (21.4)	3 (8.3)	0.35
Moderate	10 (71.4)	27 (75.0)	
Severe	1 (7.1)	6 (16.7)	
Length of stay, days	6.3 (4.0-17.5)	5.3 (3.5-10.0)	0.27
ICU admission	-	-	
**Outcomes**			
**Re-hospitalization**			
30-day	6 (40.0)	10 (23.8)	0.32
90-day	7 (46.7)	13 (31.0)	0.27
180-day	9 (60.0)	21 (50.0)	0.56
**Mortality**			
30-day	3 (20.0)	4 (9.5)	0.29
90-day	4 (26.7)	9 (21.4)	0.68
180-day	5 (33.3)	12 (28.6)	0.73

Data are presented as median (IQR), mean ±SD and n (%). Differences between groups were evaluated using t-test and Kruskal Wallis test for normal distributed variables and non-normally distributed variables, respectively. For categorical variables Pearson chi-square tests were used. P-values < 0.05 are considered significant.

Supplementary Table S6. Primary cause of re-hospitalization among undernourished, well-nourished, overweight, and obese patients after discharge from community-acquired pneumonia

**Admission cause**	Total sample (n=129)	Undernourished (n=30)	Well-nourished (n=35)	Overweight (n=30)	Obese (n=34)
Pneumonia	35 (27.1)	7 (23.3)	7 (20.0)	12 (40.0)	9 (26.5)
Pulmonary	36 (29.9)	10 (33.3)	10 (28.6)	6 (20.0)	10 (29.4)
Cardiovascular	11 (8.5)	4 (13.3)	4 (13.3)	2 (6.7)	3 (8.8)
Other	47 (36.4)	9 (30.0)	16 (45.7)	10 (33.3)	12 (35.3)
Data presented as n (%)					

Supplementary Table S7. Differences in cause of re-hospitalization among undernourished, well-nourished, overweight, and obese patients hospitalized with community-acquired pneumonia.

	**Well-nourished vs. undernourished**	**P**	**Well-nourished vs. overweight**	**P**	**Well-nourished vs. obese**	**P**	**Overweight vs. under-nourished**	**P**	**Overweight vs. obese**	**P**	**Underweight vs. obese**	**P**
Pulmonary	-3 (-54; 8)	0.89	-20 (-61; 21)	0.37	-6 (-47; 35)	0.78	17 (-25; 59)	0.45	14 (-26; 50)	0.50	-3 (-43; 38)	0.89
Pneumonia	-4 (-45; 37)	0.85	9 (-34; 52)	0.69	0 (-40; 40)	1.00	-13 (-56 (30)	0.58	-9 (-52; 57)	0.69	4 (-37; 45)	0.85
Cardiovascular diseases	-7 (-54; 40)	0.79	-1 (-0.49; 47)	0.97	-3 (-49; 43)	0.90	-6 (-54; 42)	0.82	-2 (-0.49; 0.46)	0.94	4 (-42; 50)	0.87
Other	16 (-23; 55)	0.43	13 (-25; 51)	0.51	11 (-25; 47)	0.55	3 (-39; 45)	0.89	-2 (-42; 38)	0.92	-5 (-45; 35)	0.81

Supplementary Table S8. Risk of re-hospitalization and mortality within 30, 90, and 180 days after discharge among undernourished, well-nourished, overweight (reference), and obese patients hospitalized with community-acquired pneumonia.

	Model 1 (unadjusted)		Model 2 (adjusted)	
**Re-hospitalization**	**HR (95% CI)**	p-value	**HR (95% CI)**	p-**value**
**30-day**				
Undernourished	1.5 (0.8; 2.6)	0.18	1.2 (0.7; 2.2)	0.52
Well-nourished	1.3 (0.7; 2.3)	0.36	1.3 (0.7; 2.2)	0.40
Overweight	Ref.		Ref.	
Obese	1.3 (0.7; 2.2)	0.40	1.4 (0.8; 2.5)	0.23
**90-day**				
Undernourished	1.6 (1.0; 2.7)	0.06	1.3 (0.8; 2.1)	0.37
Well-nourished	1.5 (0.9; 2.4)		1.5 (0.9; 2.4)	0.11
Overweight	Ref.		Ref.	
Obese	1.6 (1.0; 2.6)	0.054	1.8 (1.1; 2.9)	0.01
**180-day**				
Undernourished	1.9 (1.3; 2.7)	0.002	1.5 (1.0; 2.2)	0.06
Well-nourished	1.5 (1.0; 2.2)	0.06	1.4 (1.0; 2.1)	0.08
Overweight	Ref.		Ref.	
Obese	1.6 (1.2; 2	0.02	1.8 (1.2; 2.6)	0.002
**Mortality**	**OR (95% CI)**	**p-value**	**OR (95% CI)**	**p-value**
**30-day**				
Undernourished	14.8 (1.8; 123.9)	0.01	8.3 (0.9; 73.1)	0.06
Well-nourished	4.0 (0.4; 38.9)	0.24	3.7 (0.4; 38.0)	0.27
Overweight	Ref.		Ref.	
Obese*	-		-	
**90-day**				
Undernourished	7.6 (2.3; 24.6)	0.01	5.0 (1.5; 17.0)	0.01
Well-nourished	1.7 (0.4; 6.3)	0.47	1.6 (0.4; 6.2)	0.52
Overweight	Ref.		Ref.	
Obese	1.1 (0.2; 4.9)	0.94	1.4 (0.29; 6.6)	0.69
**180-day**				
Undernourished	8.7 (3.0; 25.1)	<0.001	5.4 (1.8; 16.5)	0.003
Well-nourished	2.8 (0.9; 8.5)	0.07	2.7 (0.9; 8.6)	0.09
Overweight	Ref.		Ref.	
Obese	1.7 (0.5; 6.0)	0.37	2.2 (0.6; 7.9)	0.22

*none died. Cox regression was fitted for new hospital admission within 30 and 180 days after discharge with mortality as competing event. Logistic regression was fitted for mortality within hospital, and within 30 and 180 days after discharge. Estimates shown are sub-distributed hazard ratio (HR) and odds ratios (OR) with corresponding 95% confidence intervals (CI) and p-value. Model 1: unadjusted. Model 2: adjusted for age, sex, and COPD.
